# Enhancing the Migration Ability of Mesenchymal Stromal Cells by Targeting the SDF-1/CXCR4 Axis

**DOI:** 10.1155/2013/561098

**Published:** 2013-12-05

**Authors:** Leah A. Marquez-Curtis, Anna Janowska-Wieczorek

**Affiliations:** ^1^Canadian Blood Services, Research and Development, Edmonton, AB, Canada; ^2^Department of Medicine, University of Alberta, CBS Building, 8249-114 Street NW, Edmonton, AB, Canada T6G 2R8

## Abstract

Mesenchymal stromal cells (MSCs) are currently being investigated in numerous clinical trials of tissue repair and various immunological disorders based on their ability to secrete trophic factors and to modulate inflammatory responses. MSCs have been shown to migrate to sites of injury and inflammation in response to soluble mediators including the chemokine stromal cell-derived factor-(SDF-)1, but during in vitro culture expansion MSCs lose surface expression of key homing receptors particularly of the SDF-1 receptor, CXCR4. Here we review studies on enhancement of SDF-1-directed migration of MSCs with the premise that their improved recruitment could translate to therapeutic benefits. We describe our studies on approaches to increase the CXCR4 expression in in vitro-expanded cord blood-derived MSCs, namely, transfection, using the commercial liposomal reagent IBAfect, chemical treatment with the histone deacetylase inhibitor valproic acid, and exposure to recombinant complement component C1q. These methodologies will be presented in the context of other cell targeting and delivery strategies that exploit pathways involved in MSC migration. Taken together, these findings indicate that MSCs can be manipulated in vitro to enhance their in vivo recruitment and efficacy for tissue repair.

## 1. Introduction

Ever since the identification of a rare population of multipotent nonhematopoietic cells in the bone marrow [1], which have been later termed mesenchymal stromal cells (MSCs), there has been an enormous rise in the number of publications pertaining to these cells. Research efforts have focused not only on elucidating the biological properties of MSCs but also on developing approaches for MSC-based therapies. To date there are nearly 400 clinical trials in which MSCs are investigated as therapy for a diverse range of diseases (http://www.clinicaltrials.gov/), particularly orthopedic, autoimmune, and ischemic disorders [2, 3]. Although originally isolated from bone marrow, MSCs have since been obtained from a variety of adult and neonatal tissues including placenta, umbilical cord, Wharton's jelly, and cord blood [4, 5]. Lacking unique markers, MSCs have been defined by criteria based on their propensity to adhere to plastic, expression of CD73, CD90, and CD105, and the absence of CD34, CD45, CD14, and HLA-DR and by their trilineage differentiation into adipocytes, chondrocytes, and osteocytes [6]. Despite similar phenotypic and cytological characteristics, MSCs from diverse sources exhibit significant differences in the expression of regulatory proteins involved in cell viability, engraftment, and migration [7, 8]. Because of the ability of MSCs to differentiate into bone and cartilage, their clinical application in musculoskeletal regeneration is actively being explored [3]. However, while there are claims of their potential to differentiate into hepatocytes and neurons [9, 10], solid evidence of differentiation that transcends the germ-layer boundaries remains scarce [11, 12]. The therapeutic efficacy of MSCs appears to be derived from their ability to secrete a wide array of bioactive molecules that are immune-modulatory, antiapoptotic, anti-inflammatory, proangiogenic, chemotactic, or stimulatory of tissue regeneration [13–19]. As MSC migration and recruitment are crucial to the success of MSC-based therapies, the purpose of this review is to appraise current investigations on the migration of MSCs to sites where they are needed.

Because MSCs can be found, albeit in small numbers, in almost all tissues where they play a role in maintaining homeostasis, it has been proposed that therapeutic efforts should focus on maximizing the mobilization of endogenous MSCs within patients to take advantage of their intrinsic regenerative potential [20, 21]. In this review, we will first briefly discuss how endogenous MSCs respond to signals of cellular damage and contend that therapy using endogenous MSCs for *in situ* regeneration becomes inadequate in patients with late-stage disease or severe injury and in those who are older; therefore, we will focus on administration of exogenous MSCs. Despite their innate ability to migrate to sites of injury or inflammation, the homing process of MSCs can be inefficient due to limitations in the cellular signals that regulate their trafficking. In fact, the majority of infused MSCs fail to reach the site of injury and consequently only minimal therapeutic benefit has been demonstrated in clinical trials [22, 23]. We will also summarize existing evidence of the biodistribution of MSCs after infusion based on current cell tracking techniques. As MSCs exhibit a wide array of chemokine and adhesion receptors that are involved in their migration, we will also describe cell manipulation approaches that target ligand/receptor interactions directing MSCs to sites of injury and characterize molecular signalling pathways governing MSC homing.

A number of studies have shown that the chemokine stromal cell-derived factor-1 (SDF-1, also known as CXCL12) is critical for stem/progenitor and mesenchymal cell chemotaxis and organ-specific homing in injured tissue through interaction with its cognate receptor CXC chemokine receptor 4 (CXCR4) on the surface of these cells (reviewed in [24–27]). Although CXCR4 is highly expressed by MSCs within the bone marrow, its expression is markedly reduced during ex vivo expansion of MSCs [28, 29], which decreases their ability to respond to homing signals emanating from injured sites. In this review we will describe strategies proposed by us and others to enhance the migration ability of systemically infused exogenous MSCs.

## 2. Mobilization of Endogenous MSCs versus Exogenously Administered MSCs

Tissue repair is an essential mechanism for maintaining the integrity and function of the human body as it is frequently exposed to physical insults that may damage various tissues and organs. Within minutes of injury, several types of inflammatory cells including neutrophils, monocytes, and lymphocytes rush to the damaged site and secrete a broad spectrum of cytokines and growth factors that attract other cells residing in tissue and in circulation. Endogenous MSCs are mobilized and contribute to tissue repair by acting as a reservoir of cells for regeneration [30, 31] and guarding against inflammation [32]. MSCs communicate with other cells in the human body and appear to “home” to areas of injury in response to signals of cellular damage (reviewed in [33]). MSCs themselves produce a variety of molecules that provide further cues for regenerative pathways, allowing MSCs in niches throughout the body to support other cell populations and contribute to tissue repair [12, 34, 35]. As such, endogenous MSC homing is being exploited as a therapeutic option because it does not require extensive ex vivo cell manipulation or vehicles for delivery. Approaches currently under development include use of chemokines and other chemotactic factors to recruit a sufficient number of endogenous MSC to effect tissue regeneration and/or colonize biomaterial scaffolds (reviewed in [21, 36]). However, in reality the reservoirs of MSCs can be depleted by degenerative disease, physiological aberrations, or age. In fact, it has been shown that the number of human MSCs obtained by marrow aspiration declines with age and that there is an age-related decline in overall suitability of endogenous MSCs for cell-based therapies [37]. Defects in tissue repair constitute a severe health problem and are frequently seen not only in older individuals but in patients with diabetes, those receiving chemo- or radiotherapy, and other patients whose immune system is suppressed (reviewed in [38]). In such cases, administration of exogenous MSCs could be a viable alternative to harnessing the limited number of circulating endogenous MSCs.

The route of administration of exogenous MSCs is an important factor in determining their fate. Local or site-directed delivery of MSCs has recently been shown as beneficial in certain settings, such as the intra-articular injection of autologous MSCs in patients with knee osteoarthritis [39]. Also, intramyocardial injection of MSCs has been the most widely used route of delivery into infarcted myocardium. For example, local direct intra-myocardial injections of autologous MSCs administered to patients with coronary artery disease and refractory angina have resulted in significant improvements in clinical parameters [40]. However, although this technique guarantees localised delivery to the inflamed tissue, it has restricted clinical applicability because it is invasive and can lead to cardiac arrhythmias [41].

Systemic delivery of exogenous MSCs in contrast provides not only a minimally invasive alternative, but also enables the delivery of multiple doses of ex vivo expanded cells and ensures that the cells remain in proximity to blood vessels rich in oxygen and nutrients. Systemic infusion also allows MSCs to exert their immune-modulatory effects in the context of inflammatory-mediated disorders [22, 42, 43]. It is now recognized that MSCs participate in immune surveillance and exert pleiotropic effects on both adaptive and innate immune systems [44, 45]. The first MSC-based drug approved for market, Prochymal, which consists of human bone marrow-derived MSCs, is intravenously administered to pediatric patients with acute graft-versus-host disease to modulate adverse immune and inflammatory responses [46]. However, it has been realized that during intravenous delivery there is a loss of MSCs in the vasculature, mostly in the lungs and liver [33, 47]. Alternatively, MSCs passively arrest and interrupt blood flow during the first pass through the precapillary vessels thus preventing the majority of infused MSCs from homing to damaged or diseased tissues [48]. Understanding the biodistribution of transplanted MSCs is essential for designing protocols for the efficient delivery of MSCs to sites of inflammation and injury.

## 3. Tracking the Migration of MSC

In view of the lack of MSC-specific markers, sophisticated detection systems and novel animal models are being explored for a detailed analysis of MSC homing and engraftment in vivo including *in situ* hybridization, fluorescence labelling, radioactive labelling, immunofluorescence, and bioluminescence. Green fluorescent protein (GFP) was used to track the distribution and fate of MSCs for at least 28 days after local delivery to rat hearts with myocardial infarction [49]. MSCs constitutively expressing red fluorescence were radioactively labeled with ^51^Cr and infused into mice; these MSCs were shown to have short-term survival and scarce distribution beyond the lungs [47]. In contrast, MSCs labeled with iron oxide nanoparticles and tracked by magnetic resonance imaging displayed preferential migration and subsequent engraftment in the thymus as well as the gastrointestinal tract but not the lungs [50]. In another study, MSCs labelled with ^111^In-oxine were shown to be largely localised in pulmonary capillaries and survived only transiently [51]. Bone marrow-derived MSCs from GFP transgenic male mice were transfused to female mice which were inflicted with burn wounds; thereafter, Y chromosome-positive cells were shown to be present in the injured sites by fluorescence *in situ* hybridization [52]. Thus, evidence is accumulating that some MSCs eventually reach sites of injury where they exert their potential for tissue repair.

Several studies have demonstrated beneficial effects of intravenous MSCs in a variety of disease models despite their low engraftment rate. Five out of six children with osteogenesis imperfecta had clinical improvement of 60% to 94% even though engraftment did not exceed 1% [53]. Intravenous infusion of MSCs three hours after acute myocardial infarction in mice resulted in a reduced infarct size and a slight improvement in the left ventricular function one month later, although after 24 hours only approximately 3% of injected MSCs were found mostly in the border zone of infarcted myocardium [54]. Systemic infusion of adipose-derived MSCs significantly reduced the severity of experimental arthritis even when MSCs were no longer present [55]. In mice infused with bone marrow-derived luciferase-labelled MSCs, peak luminescence persisted in the liver 24 h after reperfusion, after which luciferase activity gradually declined; nevertheless, remnant liver regeneration rate was accelerated relative to the control group [56].

Attempts have been made to enhance recruitment by increasing the dose of MSCs infused. Although the optimal dosage of MSCs for their use in therapeutic applications is still unclear and should be dependent upon the type of cell therapy, at least 1 to 2 × 10^6^ MSCs per kg of patient body weight is generally used [30]. In fact, given the low engraftment rate of MSCs, a dose ranging from 150 million to 300 million cells administered twice per week over the course of two weeks is usually used in clinical trials [57]. The high dose required for clinical application of MSCs entails extensive in vitro expansion, which has been shown to cause enlargement of cells [58]. This could explain the observation in a rat model that a large fraction of systemically infused MSCs becomes massively entrapped within the lungs and filtering organs as emboli [19, 59]. As the numbers of primary MSCs which can be isolated are too low for clinical use, it is necessary to expand MSCs in culture, and significant progress has been made to generate various culture media formulations to replace ill-defined serum and human-sourced supplements [60].

**Figure 1:**
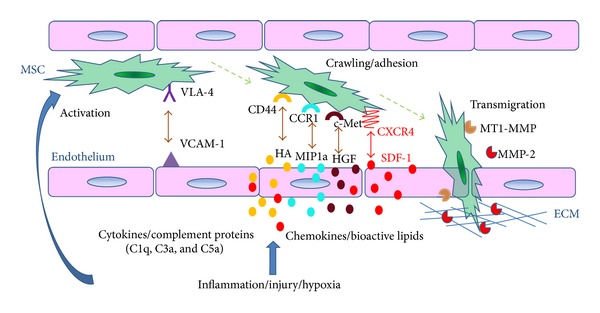
Mechanisms of MSC transendothelial migration towards injured tissue. Mesenchymal stromal cells (MSCs) express the *α*_4_ *β*_1_ integrin very late antigen (VLA)-4, and endothelial cells express the corresponding ligand, vascular cell adhesion molecule (VCAM)-1. The onset of inflammation in injured tissue causes the release of cytokines which upregulate VCAM-1 and activates VLA-4, leading to initial arrest of MSC on the endothelium surface. MSCs also express a variety of homing receptors including CXCR4, CD44, CCR1, and c-Met, and their corresponding ligands, namely SDF-1, hyaluronic acid, M1P-1(alpha), and HGF, respectively, are upregulated at the site of tissue injury and/or hypoxia. These ligand-receptor interactions, as well as chemotactic bioactive lipids, modulate cell-cell contact between MSCs and endothelia cells. In addition, complement proteins that are stimulated by inflammation such as C1q, C3a, and C5a also chemoattract MSCs. Moreover, MSCs express the extracellular matrix-degrading enzymes, matrix metalloproteinase-(MMP-) 2 and membrane type (MT)1-MMP that play a role in their extravasation.

However, the possibility exists that the MSC phenotype and biological properties vary between in vivo and in vitro settings due to removal from their natural environment and the use of chemical and physical growth conditions that might alter their characteristics. MSCs are known to undergo phenotypic rearrangements during ex vivo manipulations, losing expression of some markers while also acquiring new ones [34]. Microarray analysis revealed that gene expression profiles of ex vivo expanded MSCs were mainly influenced by the level of cell confluence rather than initial seeding density, and the genes that were upregulated in MSCs are involved in chemotaxis, inflammation, and immune responses [61]. Different cell sources and culture conditions are also likely to affect the MSC phenotype and hence their migratory patterns [7]. For example, freshly isolated, uncultured MSCs have been reported to migrate to bone marrow and spleen after systemic transplantation in experimental animal models. In contrast, culture-expanded MSCs appear unable to migrate and home to the bone marrow [57, 62, 63]. Whereas tracking studies have shown that the majority of MSCs immediately localize to the lungs after intravenous infusion, they tend to disappear from the lungs within hours and migrate to other tissues, such as the liver, spleen and kidney, and preferentially to sites of injury or tumours [51, 59, 64, 65]. Despite the fact that the endothelium is functionally different in various tissues, in general MSCs follow a multistep homing cascade (activation, adhesion and transmigration) to migrate across the endothelium, which is similar to that employed by leukocytes migrating to sites of inflammation [66–68] (Figure 1). MSCs express a variety of cell adhesion molecules and homing receptors. However, the apparent lack of MSC rolling had been attributed to the absence of selectin receptors [69]. The release of inflammatory cytokines triggers the activation of vascular cell adhesion molecule (VCAM)-1 expressed by endothelial cells and *α*_4_ *β*_1_ integrin (VLA-4) expressed by MSCs. Tissue injury and inflammation stimulate the secretion of various growth factors and chemokines such as SDF-1 which interacts with the 7-transmembrane G-coupled receptor CXCR4 on the surface of MSCs. Finally, the activation of proteases, particularly matrix metalloproteinase-(MMP-) 2 and membrane type (MT)1-MMP expressed by MSCs, facilitates transmigration across extracellular matrix in the basement membrane.

## 4. Chemotactic Factors That Promote Tropism of MSCs to Sites of Injury

MSCs show a propensity to migrate to sites of tissue injury, such as to ischemic brain [70], infarcted myocardium [25, 71], demyelinated lesions [72], pulmonary fibrosis [73], laser-induced ocular damage and injured corneal epithelium [74, 75], kidney after ischemic reperfusion [76], and injured liver [64]. MSCs have the ability to traffic into inflamed tissues; however, their migratory mechanisms need to be elucidated before this can be exploited therapeutically. The mode of recruitment of MSCs is by chemotaxis, the directional migration in response to a gradient of soluble chemoattractants (growth factors and chemokines), as well as other factors. MSCs have been reported to express various chemokines and chemokine receptors with differences in expression likely due to isolation techniques and in vitro culture conditions [25, 29, 77, 78]. In fact, analysis of chemokine receptors in short-term and long-term murine MSC cultures showed tissue culture-dependent expression of several receptors [79]. Also, ex vivo expanded human bone marrow MSCs showed differences in the gene expression patterns of factors that play a critical role in cell migration and tissue regeneration according to the seeding density and culture time [80]. Over 40 regulatory cytokines were detected in the conditioned medium obtained from murine MSCs [51].

After culturing MSCs for 48 hours, elevated levels of monocyte chemoattractant protein-1 (MCP-1), macrophage inflammatory protein-1 (MIP-1)*α*, and MIP-1*β* were found compared to control media, and MCP-1 and MIP-1*α* increased the migration of MSCs [14]. Consistent with this finding, intravenously infused allogeneic eGFP^+^ MSCs migrated preferentially toward the heart of mice overexpressing MCP-1 compared with wild-type animals, in which migration toward heart was negligible [81]. Moreover, human MSCs migrated in response to chemokines known to be expressed in the lesions of multiple sclerosis, namely, SDF-1, MCP-1, RANTES, MIP-1*α*, and IP-10 [72], and to IL-8 which is highly expressed in gliomas [82].

The growth factor receptors for bone morphogenetic protein, epidermal growth factor, transforming growth factor, fibroblast growth factor (FGF), hepatocyte growth factor (HGF), insulin-like growth factor (IGF), platelet-derived growth factor (PDGF), and vascular endothelial growth factor (VEGF), chemokine receptors for MIP, RANTES, MCP, TARC, MDC, and SDF, and receptors for tumour necrosis factor, lysophosphatidic acid, spingosine-1-phosphate, and Toll-like receptor are expressed by bone marrow-derived MSCs [36]. In particular, the CC chemokine receptors 1 and 2 (CCR-1 and CCR-2) play a crucial role in the migration and engraftment of bone marrow-derived MSCs into ischemic myocardial tissue [25]. The chemokine receptors, namely CXCR4, CX3CR1, CXCR6, CCR1, and CCR7, expressed by a minority (2%–2.5%) of human bone marrow-derived MSCs, were linked to the in vivo migratory abilities of MSCs toward murine pancreatic islets [83]. Overexpression of CCR1 in murine MSCs increased their migration to infarcted myocardium and reduced infarct size and cardiomyocyte apoptosis [84].

Previously we showed that MSCs also express c-met, the receptor for HGF [28], whose concentration is upregulated in wound areas and shown to act as chemoattractant for MSCs [28]. HGF also mediated migration of MSCs to sites of apoptotic cell death and towards HGF-expressing glioblastomas, and this observation has been exploited in gene therapy to deliver therapeutic drugs to the tumor cells [85]. In this regard, fibrin or collagen gels loaded with HGF were used as a recruitment system for endogenous MSC to facilitate wound healing [86].

MSCs also express CD44, the receptor for hyaluronic acid, which accumulates in the kidney following ischemic injury. CD44-deficient MSCs or MSCs incubated with a CD44 blocking antibody did not migrate to kidney with glycerol-induced damage; on the other hand, transfection of MSCs with CD44 restored their recruitment to injured kidney [87].

In addition to chemotactic peptides, bioactive lipids have recently been implicated in the recruitment of MSCs. For example, ceramide-1-phosphate was shown to be upregulated in damaged tissues and to provide chemotactic homing signals to bone marrow-derived MSCs [88]. MSCs have also been shown to interact with immune cells during inflammation, and these interactions may impact the way MSCs contribute to tissue repair [44, 45]. The complement cascade (activated through the classical, alternative, or lectin pathway) leads to the generation of bioactive peptides such as C3a and C5a that are responsible for chemoattracting immune cells to sites of inflammation [89]. Although it has been shown that C3a and C5a chemoattract human MSCs [90], recently we reported that the initiator of the classical pathway, complement component 1 subcomponent q (C1q) also exerts a chemotactic effect on them [91].

Microvesicles released by MSCs have also been recently appreciated for their role in recruiting MSCs and tissue repair [18, 92, 93]. These membrane-derived microvesicles have the ability to transfer lipids, nucleic acids, and proteins (e.g., receptors such as CXCR4) to neighboring cells, thereby mediating a variety of biological responses such as self-renewal, differentiation, adhesion, and migration [93, 94]. The SDF-1/CXCR4 axis has been shown to be significantly upregulated in many experimental models of tissue injury such as myocardial infarction [95–97], ischemic brain lesion [70], acute kidney injury [98], and burn wounds [52].

## 5. The SDF-1/CXCR4 Axis in MSC Migration

It is generally believed that SDF-1 mediates cell migration through its binding with CXCR4. Therefore, the enhanced secretion of SDF-1 at the site of injury creates an environment facilitating the homing of circulating CXCR4-positive cells. MSCs stimulated with SDF-1 showed 30 differentially expressed genes, 11 of which are involved in cellular movement [99]. The SDF-1/CXCR4 pathway is crucial in the migration of MSCs to bone fractures, as MSC recruitment was abrogated when SDF-1 signalling was impaired [100]. CXCR7, another chemokine receptor, has also been shown to bind SDF-1 with high affinity [101]; however, the activation of CXCR7 by SDF-1 does not contribute to cell chemotaxis [102, 103]. Consistently, MSC migration to ischemic kidney was shown to be mainly CXCR4-dependent, as confirmed by the increased migration of CXCR4-transduced MSCs and full inhibition of MSC migration using the CXCR4 antagonist AMD3100 [98, 104]. This is in contrast to a report showing that the overexpression of CXCR4 did not improve MSC homing in a mouse model of cisplatin-induced acute kidney injury [105]. More evidence is emerging on the important role of the SDF-1/CXCR4 in the recruitment of MSCs into injury sites in animal models [24, 26, 27, 52, 95, 97, 104].

GFP-labelled MSCs were transplanted intravenously with or without treatment with CXCR4-blocking antibody into rats that had unilateral mandibular distraction osteogenesis, and their distribution in the soft callus was examined after 24 h. SDF-1 facilitated the migration of MSCs both in vitro and in vivo, and this migration was inhibited by AMD3100 [106]. Furthermore, migration to burn wounds in mice promoted the epithelialization of the wound, whereas pretreatment of the MSCs with AMD3100 attenuated wound closure [52]. In mice with acute myocardial infarction, the local trophic effects of infused MSCs required cardiac myocyte (CM)-CXCR4 expression and were mediated by SDF-1 secretion. In the absence of CM-CXCR4 expression, there was a significant loss of functional benefit in MSC-mediated repair despite equal increases in vascular density [71]. In another study, MSCs were transduced using lentiviral vectors to overexpress the CXCR4-eGFP fusion protein and injected into rats following a left middle cerebral artery occlusion. The number of eGFP-positive cells surrounding the occlusion areas was significantly increased in the CXCR4-MSC-treated group compared to the control group; moreover, they showed an increase in the capillary vascular volume of the peri-infarct area, a reduction in the volume of the cerebral infarction, and improved neurological function [107]. Taken together, these studies confirm that the interaction between locally produced SDF-1 and its receptor CXCR4 expressed on the surface of MSCs plays a crucial role in the homing of transplanted cells. However, culture-expanded MSCs show a downregulation of surface expression of key homing receptors including CXCR4 [29, 108] and their ability to respond to homing signals. Hence various strategies have been explored and employed to enhance the expression of CXCR4 in MSCs.

## 6. Preconditioning or Engineering MSCs before Infusion to Improve Cell Migration

Several groups have attempted to develop alternative approaches to optimize MSC migration efficiency and potentiate their beneficial effect at the site of the injury by preconditioning MSCs before infusion with various compounds (e.g., drugs and/or growth factors) possessing pro-survival and pro-migratory properties, by certain physical treatments (e.g., hypoxia) (reviewed in [109]), or by cellular modification (reviewed in [110]). Specifically, MSCs have been conjugated with ligands (e.g., Sialyl Lewis^x^) that mediate cell rolling, adhesion, and transmigration to inflamed tissue [111]. Also, coating MSCs with antibodies against vascular cell adhesion molecule and addressins enhanced their delivery to inflamed colon in a mice model of inflammatory bowel disease [112]. Genetically modified MSCs expressing P-selectin glycoprotein ligand −1 and Sialyl-Lewis^x^ exhibited robust rolling leading to increased homing in injured ear; further transfection with IL-10 significantly improved the anti-inflammatory impact of these cells [113].

In recognition of the fact that aging reduces the number of MSCs that can differentiate into osteoblasts, leading to impairment of osteogenesis, it has been proposed that if MSCs could be directed toward the bone surface, they could be a viable therapeutic option for bone regeneration. To this end MSCs have also been conjugated to a synthetic peptidomimetic ligand (LLP2A) that has high affinity for activated *α*_4_ *β*_1_ integrin in order to direct the transplanted MSCs to bone. This treatment significantly increased the rate of bone formation and restored both trabecular and cortical bone loss induced by estrogen deficiency or advanced age in mice [114].

Preconditioning with insulin-like growth factor-(IGF-) 1 improved the migration capacity of MSCs and restored normal renal function after acute kidney injury through a mechanism involving the upregulation of CXCR4. Its functional role was further confirmed by blocking CXCR4 which totally abrogated the pro-migratory effect of IGF-1 on MSCs [115]. In a rat model of myocardial infarction MSCs treated with a combination of IGF-1, fibroblast growth factor-2 and bone morphogenic protein-2 showed enhanced expression of cardiac transcription factors [116]. In a mouse model of osteogenesis imperfecta, priming human fetal MSCs with SDF-1 upregulated surface CXCR4 expression and enhanced in vitro chemotaxis, which translated to increased in vivo engraftment in the bone and bone marrow, improved bone quality and plasticity, and a trend to lower fracture rate [117].

Treatment with inhibitors of glycogen synthase kinase-3*β* (GSK-3*β*) increased the levels of CXCR4 and matrix metalloproteinase (MMP)-2 and membrane type (MT)1-MMP [118], which we have shown to be involved in MSC migration [28]. In another study, EPO combined with G-CSF enhanced MMP-2 expression in MSCs, promoted MSC motility, and activated the extracellular signal related kinase (ERK)1/2 signaling pathway [119]. Preconditioning MSCs with oxytocin (OT) have also been proposed as a novel strategy for enhancing therapeutic potential of these cells in ischemic heart because, following OT treatment, MSCs respond with rapid calcium mobilization and upregulation of the protective protein kinase B (PKB or Akt), phospho-ERK1/2 proteins and genes with angiogenic and antiapoptotic properties, such as vascular endothelial growth factor, thrombospondin, tissue inhibitors of metalloproteinase-(TIMP-) 1, TIMP-2, TIMP-3, and MMP-2 [120]. In a related study, two constituents of traditional Chinese herbal medicine (tanshinone IIA and astragaloside IV) were shown to increase CXCR4 expression, in vitro migration, and the capacity of MSCs to home to rat ischemic myocardium [121]. Previously, we reported that a histone deacetylase inhibitor, valproic acid (VPA), increases CXCR4 expression on CD34+ HSPCs derived from cord blood and their migration towards an SDF-1 gradient [122]. We therefore investigated the effect of VPA on the migration of MSCs as we will discuss below.

Various pathologies compromise tissue reperfusion and thereby decrease tissue oxygen tension. Based on the fact that the bone marrow niche has a low oxygen tension (1–7%), hypoxic pre-conditioning during ex vivo expansion has been widely studied and shown to also have a beneficial effect on stem cell migration. Several reports have shown that short-term exposure of MSCs to hypoxia could increase their migration. The migration capacity of MSCs was improved at very low oxygen concentrations (1%) via upregulation of hypoxia-inducible factor-1*α* [123]. Hypoxic-treated MSCs were selectively recruited to ischemic kidneys in response to SDF-1*α*, and this increased chemotaxis was blocked by an anti-CXCR4 antibody but not by an anti-CXCR7 antibody [102]. Lastly, human MSCs grown under low O_2_ (5%, hypoxic) demonstrated markedly higher targeted migration activity towards wound healing cytokines such as those found in ischemic and myocardial infarcts compared to those grown under normal O_2_ (21%, normoxic) [124].

Aside from molecular and physiological conditioning, physical stimuli have also been exploited to direct the migration of MSCs. As naturally occurring electric fields exist during healing at sites of bone fracture, by culturing MSCs in direct electrical current MSC migratory response was increased [125]. Pulse-focused ultrasound has also been shown to upregulate chemoattractants and enhance the homing, permeability, and retention of human MSCs [126]. Low-level shear stress has been employed to induce MSC migration in a wound model through upregulated secretion of SDF-1 and increased CXCR4 expression [127]. Similarly, mechanical stretching of skin induced temporal upregulation of SDF-1*α* and increased the in vitro and in vivo migration of MSCs, which was significantly blocked by AMD3100, confirming the crucial role of the CXCR4 receptor [128]. In this regard, several approaches have been explored to enhance the expression of CXCR4, and we describe below the strategies employed by our group.

## 7. In Vitro Approaches to Enhance MSC Migration through CXCR4 Upregulation

### 7.1. IBAfect-Transfection of CXCR4

Currently, the most widely used method to transfer genes into MSCs is through viral delivery vectors, and several groups have attempted to overexpress CXCR4 on MSCs in this manner. Adenoviral, retroviral, and lentiviral transduction of CXCR4 have been shown to increase mobilization and engraftment of MSCs in animal models of injury [107, 129–131]. However, there is increased interest in developing safe and efficient nonviral gene delivery systems to reduce the risks of mutagenesis due to the random integration of viral genes into the host genome and the immunogenicity of the virus itself. Nucleofection has been proposed as a technique that combines an easy protocol with high effectivity and basically allows in vivo application according to Good Manufacturing Practices guidelines [132].

**Figure 2:**
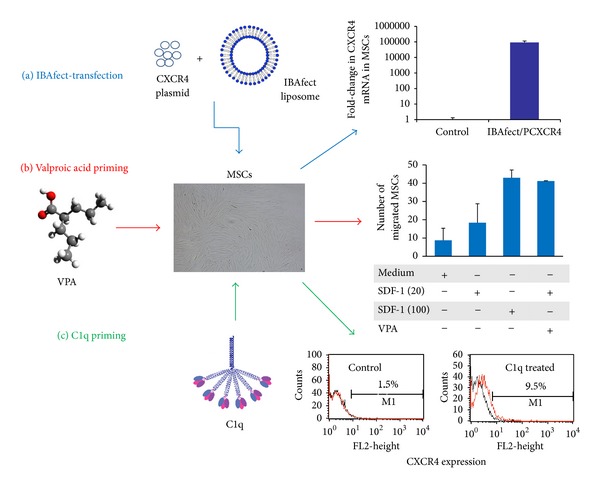
Approaches to enhance MSC migration by upregulating CXCR4. Cord blood-derived mesenchymal stromal cells (MSCs) were subjected to three treatments. (a) Transfection with CXCR4 plasmid using the liposomal reagent IBAfect led to a 10^5^-fold increase in CXCR4 mRNA expression. (b) Priming MSCs with 5 mM valproic acid (VPA) increased trans-Matrigel chemoinvasion towards a low SDF-1 gradient (20 ng/mL) to a level comparable to that of untreated cells migrating towards a high SDF-1 gradient (100 ng/mL). (c) Exposure of MSCs to 10 *μ*g/mL C1q also primed/enhanced trans-Matrigel migration towards SDF-1 and this was accompanied by over 6-fold increase in the surface expression of CXCR4 in C1q-treated cells.

Cationic liposomal delivery has emerged as another viable alternative to gene therapy using viral vectors because of its low toxicity, lack of immunogenicity after in vivo administration, low cost, and relative ease in creating nucleic acid/liposome complexes on a large scale for clinical use [133]. Previously, we reported CXCR4 gene delivery into human HSPCs using IBAfect, a polycationic liposomal transfection reagent, and achieved up to 20% transfection [134]. The specifically designed molecular structure of IBAfect ensures easy entry of plasmid DNA into cells by forming a compact complex with the lipid. We found that IBAfect-mediated CXCR4 transfection of cord blood-derived MSCs resulted in a 10^5^-fold increase in CXCR4 transcript number (Figure 2(a)), a 40% transfection efficiency, and an over 3-fold increase in chemotactic index [135]. In comparison, adenoviral transduction of rat bone marrow-derived MSCs resulted in 95% CXCR4 expression but only <2-fold enhancement in SDF-1-directed cell migration [129]. In another study, rat MSCs that were retrovirally transduced with CXCR4 showed 54% expression of CXCR4 and an approximately 3-fold increase in migration towards an SDF-1 gradient compared to non-transduced cells [130]. On the other hand, despite a significant overexpression (93%) and functionality of CXCR4 in human MSCs, no additional improvement of basal cell migration was observed [132]. CXCR4 transfection of MSCs using IBAfect presents a more efficient approach to improving the therapeutic efficacy of MSCs; however, it needs to be tested in vivo.

Despite the vital role of SDF-1-directed migration, it is limited by the relatively short half-life of SDF-1 and the highly time-dependent nature of the homing of progenitor cells to the site of injury [136]. For example, myocardial SDF-1 expression was increased only during the early post-infarct phase, and as a result only MSCs intravenously infused in temporal vicinity to the early phase of myocardial infarction were recruited to injured myocardium, enhancing angiogenesis and improving cardiac function; further, MSCs injected when the cardiac SDF-1 expression had already fallen did not home to the heart or have a positive effect on the outcome [137]. Attempts to deliver SDF-1, on the other hand, are complicated by rapid diffusion of the chemokine away from the intended site and by enzymatic degradation. SDF-1 is degraded by dipeptidyl peptidase IV [138–140] and by MMP-2 and MT1-MMP [141], which are activated at the injured site. These findings raise the need for ways to enhance or prime migratory responses to the low physiological levels of SDF-1.

### 7.2. Priming SDF-1 Homing Responses with Valproic Acid

VPA (also known as 2-propylpentanoic acid) is an anticonvulsant and mood-stabilizing drug approved by the Food and Drug Administration for the treatment of epilepsy and manic disorders. Because in rat MSCs short-term (3 h) VPA treatment more robustly enhanced CXCR4 transcript levels compared to long-term (24 h or longer) treatment [142], we exposed human cord blood-derived MSCs to VPA for 3 or 6 h. We found that increasing doses of VPA up to 10 mM enhanced CXCR4 mRNA expression to levels over 40-fold after 3 h and about 60-fold after 6 h exposure. However, while no upregulation was observed in the surface expression of CXCR4, total CXCR4 protein level was increased. It has been previously shown that CXCR4 is mostly sequestered intracellularly [143] and forms heterodimers with CXCR7 [144]. Therefore it is plausible that an increase in protein expression may not be detectable on the surface of cells. Cord blood-derived MSCs migrated about 2-fold more towards a low SDF-1 gradient (20 ng/mL) and 4-fold more towards a high SDF-1 gradient (100 ng/mL) compared to medium alone. When the cells were pretreated with 5 mM VPA for 3 h we found that VPA significantly increased trans-Matrigel chemoinvasion towards a low SDF-1 gradient to a level comparable to the chemoinvasion of untreated cells migrating towards a high SDF-1 gradient (Figure 2(b)). This priming effect of VPA on chemoinvasion towards a low SDF-1 gradient was significantly inhibited by AMD3100, supporting the hypothesis that the increase in chemoinvasion is due to a direct effect of VPA on CXCR4 expression (manuscript submitted).

The ability to cross the endothelium and degrade the extracellular basement membrane matrix is another essential step for MSCs to reach target tissues. Previously we and others showed that MMP-2 is involved in the migration of MSCs [28, 65, 145, 146]. We found that VPA increases MMP-2 gene expression and the secretion and activation of pro-MMP-2. Consistently, active MMP-2 is secreted at sites of MSC invasion in myocardial tissue [67].

Based on our findings, we proposed that short-term exposure (3 h) of MSCs to a low dose of VPA (≤5 mM) could be used to increase their recruitment to sites of injury. Although our study examined priming of MSC migratory responses in vitro, the ability of VPA to promote MSC homing and to improve functional recovery was also assessed in vivo using a rat model of cerebral ischemia [147]. MSCs primed with VPA (2.5 mmol/L, 3 h) were transplanted into rats 24 h after transient middle cerebral artery occlusion (MCAO). Priming with VPA increased the number of MSCs homing to the cerebral infarcted regions. MCAO rats receiving VPA-primed MSCs showed markedly improved neurological score, reduced infarct volume, and increased microvessel density in the infarcted penumbra regions. These beneficial effects of VPA priming were reversed by AMD3100 [147]. VPA was shown to enhance MSC migration by upregulation of the histone deacetylase-CXCR4 and glycogen synthase kinase-3*β*-MMP-9 signaling pathways [142]. Because VPA robustly improved the homing efficacy of MSC in vivo, it is likely that fewer MSC would be required to achieve clinical efficacy, thus reducing the risk of cerebral flow interruption and shortening the time necessary to culture MSCs for transplantation.

### 7.3. Priming with C1q

The initiator of the classical pathway, C1, consists of C1q and two other catalytic subunits C1r and C1s. C1q binds to its specific cell-surface receptors (namely, CD93, CR1, gC1qR, and cC1qR/calreticulin) to induce a variety of cellular responses [148]. We have previously shown that C1q enhances the homing-related responses of HSPC through binding to CD93 [149]. In a more recent study we found that C1q chemoattracts MSCs across reconstituted basement membrane in a dose-dependent manner, and MSCs pretreated with C1q sense better the SDF-1 gradient [91]. C1q increases the chemoinvasion of MSCs towards a low concentration of SDF-1 (20 ng/mL) to a level equivalent to that of a high concentration (100 ng/mL) by increasing the CXCR4 surface expression from 1.5% to 9.5% (Figure 2(c)). Although only a small increase in the percentage of the cells expressing surface CXCR4 was observed, this difference is of biological significance and is enough to elicit a homing response of MSCs consistent with previous studies showing that a slight increase in the percentage of CXCR4-expressing MSCs (e.g., induced by short-term hypoxia) results in significant improvement of migration capacity [150]. Furthermore, we demonstrated that C1q induces the secretion of MMP-2, which contributes to the migration of MSCs across reconstituted basement membrane.

Similar to the signals induced by C3a and C5a [90], the ERK1/2 signalling pathway was shown to be involved in the migration of MSCs towards C1q as PD98059, a specific ERK inhibitor, significantly inhibited C1q-directed migration of MSCs [91]. Elucidation of the signalling pathways mediating MSC migration is a crucial step towards designing MSC-based therapies. A wide array of signalling pathways has been implicated (reviewed in [151]) and a brief summary follows.

## 8. Signalling Pathways in MSC Migration

Several phosphoinositide 3-kinase (PI3K)/protein kinase B (Akt) stimulators including SDF-1 have been shown to increase MSC migration [152]. SDF-1-enhanced MSC migration was mediated through the activation of the PI3K/Akt pathway [26]. Hypoxia conditioning has also been shown to increase MSC migration via the PI3K/Akt pathway, and this effect was abolished by the PI3K inhibitors wortmannin and LY294002 [153]. The levels of phospho-Akt and phosphomitogen-activated protein kinase (MAPK) reached the maximum in the gene-modified CXCR4-MSCs and were restored to the basal level by AMD3100. Treatment of MSCs with PI3K-specific inhibitor (LY294002) and MAPK inhibitor (PD98059) significantly attenuated the migration of the CXCR4-MSCs [98]. It is likely that both PI3K/Akt and MAPK/ERK transduction pathways are involved in the enhancement of MSC migration induced via CXCR4 overexpression. In fact, MSC migration was inhibited by AMD3100, LY294002, PD98059, and p38MAPK inhibitor (SB203580). Small interfering RNA-mediated knockdown of Akt, ERK, and p38 blocked SDF-1-induced MSC migration [154].

The involvement of a Rho-kinase (ROCK) was also demonstrated using the ROCK inhibitor Y27632 which effectively promoted MSC transendothelial migration; conversely, LY294002 blocked it. Consistently, adenovirus-mediated interference of ROCK in MSCs significantly increased transendothelial migration while overexpression of a PI3K dominant negative mutant in MSCs blocked transendothelial migration [155]. The accumulation of MSCs in the site of myocardial infarct was blocked by LY294002 and phosphorylated Akt was highly increased in SDF-1-treated MSCs [97]. Pharmacological inactivation of AKT2 but not AKT1 significantly decreased cell migration and invasion [156]. Taken together, these results demonstrate the roles of PI3K, Akt, ERK, and p38 signal transduction pathways in SDF-1-directed migration of MSCs.

Lastly, Notch signalling was reported to regulate MSC migration and function, at least partially via the modulation of CXCR4 expression. The gene, protein, and cell surface expression of CXCR4 were significantly increased in MSCs when Notch signalling was interrupted by *γ*-secretase inhibitor (GSI) or by knockout of the transcription factor RBP-J, the mediator of Notch signalling. The GSI-treated or RBP-J deficient MSCs showed stronger migration toward SDF-1 and toward hepatic ischemia/reperfusion injury than that of control MSCs [157].

## 9. Concluding Remarks

The prevalence of MSC-based clinical therapies calls for rigorous investigations of their efficacy. As with any cellular therapy, national and international health regulatory agencies also typically require thorough validation of toxicology and safety in preclinical animal models, in addition to a demonstration of potency. The use of MSCs in most clinical applications, whether autologous or allogeneic, involves the isolation of cells from various tissues, followed by their ex vivo expansion; however, the lack of standardized cell culture conditions results in heterogeneous populations of cells despite proposed criteria defining characteristics of MSCs and measures of efficacy [6, 158, 159]. In addition, the production of safe MSC products requires compliance with Good Manufacturing Practices to ensure that they are free of any contamination. Recently, the safety of MSCs, particularly in regard to their genetic stability in long-term expansion, cryopreservation, and banking, and the role of serum in cultures, as well as the intravascular delivery of MSCs, was reviewed, and it was concluded that the vast majority of clinical trials conducted with MSCs have not reported major health concerns [160, 161]. Malignant transformation of infused MSCs has been refuted as these cells did not demonstrate sustained engraftment [162]. Strategies to effectively direct MSCs to sites of injury and inflammation including genetic manipulation, modifying expression of homing and adhesion receptors, and antibody or peptide-directed cell targeting [110] are actively being pursued. It is likewise essential to ensure that these manipulations of MSCs do not alter their overall genotype, phenotype, functional potential, and other biological characteristics. For example, a possible cause of the failure of MSC-based immunotherapy in clinical trials has been attributed to impairment of immunosuppressive properties of cryopreserved MSCs [23, 163]. An understanding of the mechanisms of MSC migration, as part of the scientific assessment of the pharmacology of MSC-based therapies, is of paramount importance. Modulation of the homing properties of MSCs could allow for their efficient recruitment to sites of injury and inflammation and could reduce the dose of infused cells required, potentially limiting the cost of this therapy as well as the risks of transformation during culture expansion.
